# Transpapillary tissue sampling of biliary strictures: balloon dilatation prior to forceps biopsy improves sensitivity and accuracy

**DOI:** 10.1038/s41598-020-74451-9

**Published:** 2020-10-15

**Authors:** Daniel Pörner, Dominik J. Kaczmarek, Dominik Heling, Annekristin Hausen, Raphael Mohr, Robert Hüneburg, Hanno Matthaei, Tim R. Glowka, Steffen Manekeller, Hans-Peter Fischer, Marieta Toma, Jacob Nattermann, Christian P. Strassburg, Maria A. Gonzalez-Carmona, Tobias J. Weismüller

**Affiliations:** 1 Department of Internal Medicine I, University Hospital Bonn, Venusberg-Campus 1, 53127 Bonn, Germany; 2 Department of Surgery, University Hospital Bonn, Venusberg-Campus 1, 53127 Bonn, Germany; 3 Institute of Pathology, University Hospital Bonn, Venusberg-Campus 1, 53127 Bonn, Germany

**Keywords:** Bile ducts, Bile duct cancer, Pancreatic cancer, Gastroenterology, Bile ducts

## Abstract

The early and definitive diagnosis of malignant bile duct stenoses is essential for a timely and adequate therapy. However, tissue sampling with transpapillary brush cytology (BC) or forceps biopsy (FB) remains challenging. With this study, we aimed to compare the effectiveness and safety of different tissue sampling modalities (BC, FB without/after previous balloon dilatation). Standardized database research identified all patients, who underwent endoscopic retrograde cholangiography with BC and/or FB for indeterminate bile duct stenosis between January 2010 and April 2018 and with a definitive diagnosis. 218 patients were enrolled (149 cases with malignant and 69 with benign disease). FB had a significant higher sensitivity than BC (43% vs. 16%, p < 0.01). Prior balloon dilatation of the stenosis improved the sensitivity of FB from 41 to 71% (p = 0.03), the NPV from 36 to 81% (p < 0.01) and the accuracy from 55 to 87% (p < 0.01). The complication rates did not differ significantly between the modalities. In our center FB turned out to be the diagnostically more effective procedure. Balloon dilatation of the stenosis before FB had a significant diagnostic benefit and was not associated with a higher complication rate.

## Introduction

Biliary strictures are frequently caused by malignant tumors of the intra- or extrahepatic biliary tree, the gallbladder, the liver, the pancreas or the surrounding lymph nodes or by metastasis in these organs. However, benign conditions like autoimmune or infectious diseases or posttraumatic ischemias can also induce inflammation and scarring fibrosis, that mimic a malignant stricture. Unless the underlying history and the typical localization and configuration of the stricture unambiguously indicate a benign etiology, we speak of an indeterminate biliary stricture (IBiS), which is highly suspicious for malignancy but lacks a confirmative tissue diagnosis^1^. This is often the case in patients with primary sclerosing cholangitis (PSC), who develop fibrotic bile duct strictures but have a risk of 1.4% per year^2^ and a life-time risk of up to 14%^3^ to develop hepatobiliary malignancies, and in patients with chronic pancreatitis, who frequently develop strictures due to inflammation of the pancreatic head but also have an increased risk of pancreatic cancer (PCA)^4^.

The early and definitive exclusion or securing of malignancy of bile duct strictures is essential in order to avoid overtreatment (e.g. non-indicated surgical interventions or non-indicated chemotherapies) on the one hand, but on the other hand to allow for a curative resection or at least an immediate start of a palliative chemotherapy.

Current guidelines^5,6^ emphasize the importance of additional imaging like endoscopic ultrasound (EUS), computed tomography (CT) or magnetic resonance imaging (MRI) for the characterization and, if necessary and possible, targeted biopsy of a stricture-related mass. Furthermore, a potential benefit of additional endoscopic tools like cholangioscopy, intraductal ultrasound or laser endomicroscopy is discussed. Despite these methods, however, it is still the primary goal to obtain enough tissue for histological or cytological diagnosis. Since endoscopic retrograde cholangiography (ERC) is indicated under most circumstances, transductal brush cytology (BC) and transpapillary forceps biopsy (FB) are the methods of choice for tissue sampling but both with limited overall sensitivity. In the present study we aimed to identify the best diagnostic strategy considering relevant co-variates (type of forceps, localization of stricture, previous intervention) and complications.

## Methods

**Figure 1:**
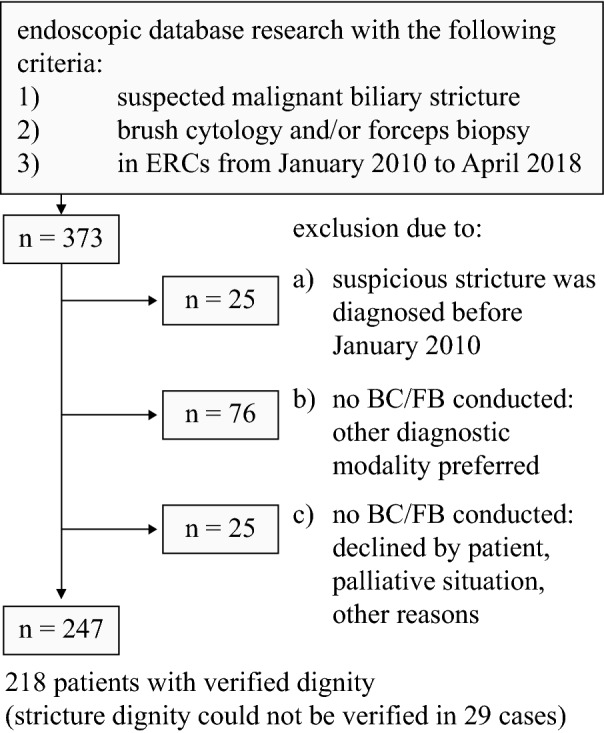
Patient enrollment. *ERC* endoscopic retrograde cholangiography, *BC* brush cytology, *FB* forceps biopsy.

**Figure 2:**
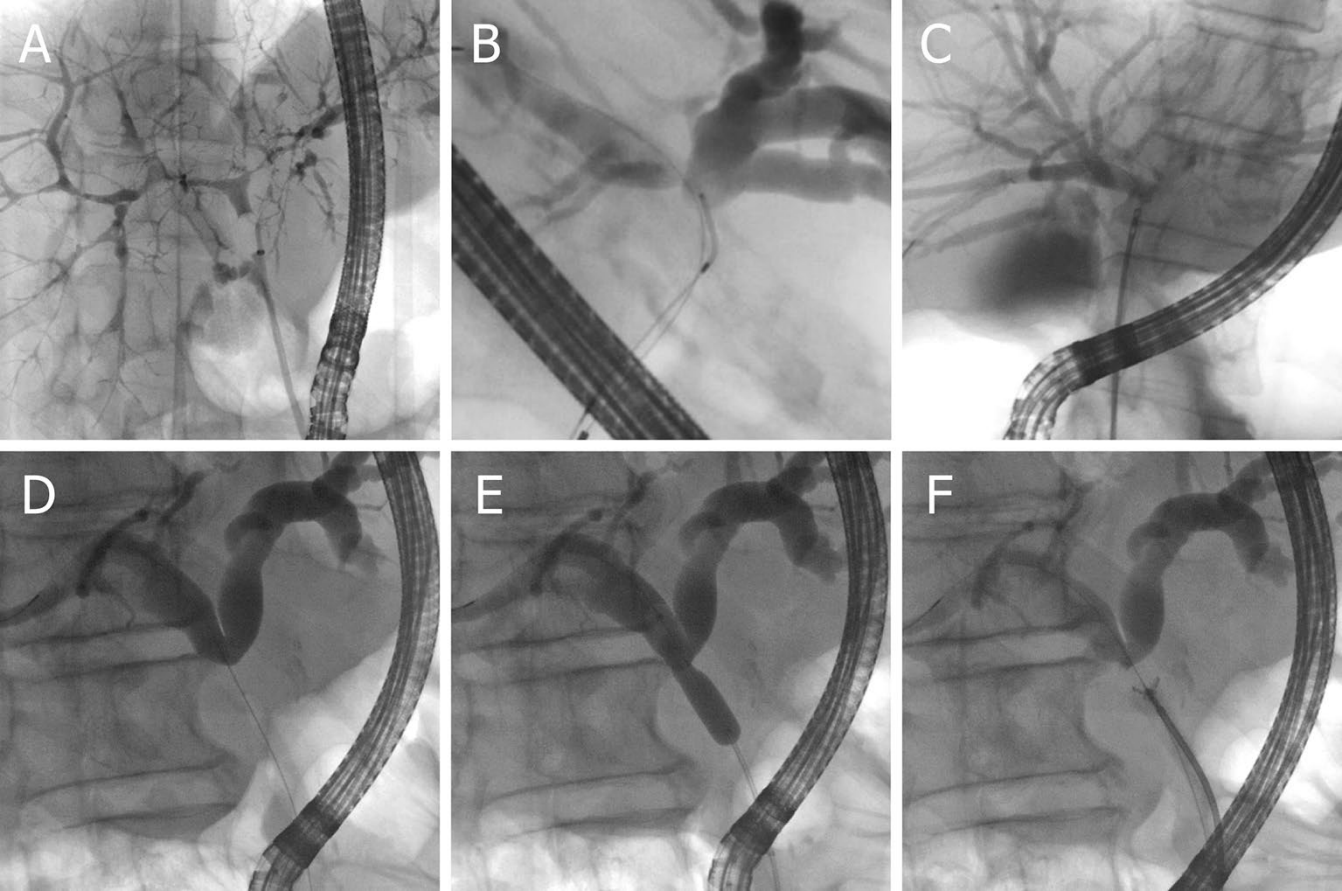
Biliary strictures and different modalities for tissue sampling. (**A**) Dominant stricture of the left intrahepatic duct in a patient with advanced primary sclerosing cholangitis. (**B**) Acquisition of a brush cytology of a hilar stricture. (**C**) Small diameter forceps biopsy of a hilar stricture. (**D**–**F**) Cholangiography revealed a highly suspicious stricture of the proximal common bile duct in a patient undergoing endoscopic retrograde cholangiography due to obstructive jaundice. After a guide-wire had been passed over the stricture (**D**), balloon dilatation of the stricture was performed (**E**). Subsequently, multiple specimens were obtained using a large diameter forceps (**F**). Histopathologic examination exposed the stricture to be caused by a cholangiocarcinoma.

Through a structured database query of our endoscopy documentation system (Viewpoint 5, GE Healthcare) we identified all patients, who were first diagnosed with a biliary stricture suspicious for malignancy between January 2010 and April 2018 and underwent ERC with BC and/or FB at the University Hospital Bonn (Fig. 1). Strictures were considered suspicious for malignancy based on patient’s history and symptoms (e.g. painless jaundice, history of PSC, no previous surgery/trauma) and/or cholangiographic features including an irregular margin, asymmetric or abrupt narrowing and the double-duct sign (see Fig. 2A).

Choice and order of the diagnostic tools (BC and/or FB) depended on the assessment and preference of the respective endoscopist. For BC we used a 6 Fr cytology brush (Cook Medical Fusion Cytology Brush, length 25 mm, Fig. 2B). For FB, two different forceps types were used: Larger forcepses (Endo-Flex Biopsy Forceps, outer diameter 2.3 mm, oval cups and spike) were preferably used for strictures of the common bile duct (CBD) or the hilar region and were positioned free-handed under fluoroscopic control (Fig. 2F). For strictures of the intrahepatic ducts, usually smaller forcepses (MTW Biopsy Forceps, outer diameter 1.8 mm, oval spoon-shaped mouth without spike) were advanced through a 10 Fr pushing catheter (Endo-Flex Pusher, Fig. 2C) as described previously^7^. Strictures were frequently dilated with a balloon (Cook Medical Fusion Titan Biliary Dilation Balloon, 4–10 mm) before or after taking 1–3 biopsies (Fig. 2D–F). In most cases, biliary stents (Boston Scientific Flexima endoprostheses, 7–11.5 Fr; Endo-Flex double-pigtail stents, 7 or 10 Fr) were placed to secure bile flow.

We retrospectively recorded patient characteristics (i.e. age, gender, pre-existing diseases, prior surgery involving the hepatobiliary tract, previously diagnosed malignoma), ERC procedure details (i.e. modalities for tissue sampling including chronological order, previous dilatation, sphincterotomy or stenting, duration of ERC), stricture characteristics (localization, length, extent), ERC-induced complications (pancreatitis, cholangitis, hemorrhage with requirement of blood transfusions, perforation) and pre-interventional CA 19-9 levels.

Final diagnosis of malignancy was based on (a) cytologic and/or histologic evidence obtained by tissue sampling during ERC, endoscopic or percutaneous fine needle biopsy (FNB), surgery or autopsy; or (b) clinical course during a follow-up of at least six months. Definitely malignant and severely suspicious cyto-/histopathological findings were classified as positive. Nearly all tissue samples acquired via BC or FB were examined by two of our local pathologists with appropriate expertise. In cases when BC and FB were conducted during the same ERC, tissue samples from both modalities were examined by the same pathologists. A stricture was considered benign when there was no evidence for malignancy during a follow-up of at least 6 months (i.e. absence of radiomorphologic tumor progression including infiltration and/or metastatic dissemination). Unless otherwise specified, the following analyses are based on each patient’s first ERC in domo that fulfills the above mentioned inclusion criteria. The study was performed in accordance with the Declaration of Helsinki (as revised in 2013) and was approved by the Ethics Committee of the Medical Faculty of the University of Bonn (approval number 233/17). Written informed consent for ERC including tissue sampling and stricture dilatation was obtained from all patients.

### Statistical analysis

Categorical variables are expressed as numbers with percentages in parenthesis. Continuous data are presented as mean ± standard deviation. Statistical comparison of proportions was performed using Chi^2^ test (with Yates correction) and two-tailed Fisher’s exact test, respectively. Unpaired *t* test with Welch’s correction was used to compare means of continuous variables. A p-value less than 0.05 was considered statistically significant. Statistical analyses were performed with IBM SPSS 25.

## Results

### Patients’ characteristics and final diagnosis

Endoscopic database research yielded a total of 247 patients with IBiS, who underwent ERC from January 2010 to April 2018 and received further sampling with BC and/or FB (Fig. 1). No definitive diagnosis could be obtained in 29 cases due to an insufficient follow-up, i.e. less than 6 months (for death or other reasons). Therefore, 218 patients (Table 1) remained for the final statistical analysis.

**Table 1:** Patients’ characteristics and pre-existing conditions.

	Benign stricture (n = 69)	Malignant stricture (n = 149)	All strictures (n = 218)
Age (years)	52.50* ± 17.26	68.54* ± 12.02	63.46 ± 15.74
Sex (female/male)	26/43	60/89	86/132
Mean follow-up (months)	28.52* ± 21.16	13.30* ± 14.30	18.12 ± 18.17
Pre-interventional CA 19-9 (U/ml)	92.95* ± 365.98	4218.72* ± 11,828.27	3081.70* ± 10,222.82
**Pre-existing conditions/prior surgery of the hepatobiliary tract**			
PSC	35/69*	5/149*	40/218
SSC/IAC	6/69*	2/149*	8/218
Chronic pancreatitis	6/69	4/149	10/218
Choledocholithiasis	20/69*	17/149*	37/218
Prior cholecystectomy	17/69	37/149	54/218
Cholecystolithiasis	10/49^†^	28/107^†^	38/156^†^
Prior partial hepatectomy	2/69	2/149	4/218
Previously diagnosed extrahepatobiliary malignoma	14/69	42/149	56/218

*PSC* primary sclerosing cholangitis, *SSC* secondary sclerosing cholangitis, *IAC* IgG4-associated cholangitis.

*p < 0.05 for comparison benign vs. malignant strictures, ^†^presence of cholecystolithiasis unknown in 3 cases with benign stricture and 5 cases with malignant stricture.

Malignant disease was finally diagnosed in 149 patients. Final malignant diagnosis based on (a) cytological and/or histological evidence obtained by surgery/autopsy (n = 81), percutaneous tissue sampling (n = 18), EUS-FNA (n = 2), tissue sampling via BC/FB (n = 38) and (b) the clinical course during a follow-up of 6 months (n = 10, mean follow-up for this cohort: 12.59 ± 7.10 months). Cases with confirmation of malignancy by multiple diagnostic modalities were subsumed under the first mentioned method. Benign condition was finally diagnosed in 69 patients by (a) surgery (n = 24) and (b) the clinical course during a follow-up of at least 6 months (n = 45, mean follow-up for this cohort: 27.15 ± 18.10 months).

**Figure 3:**
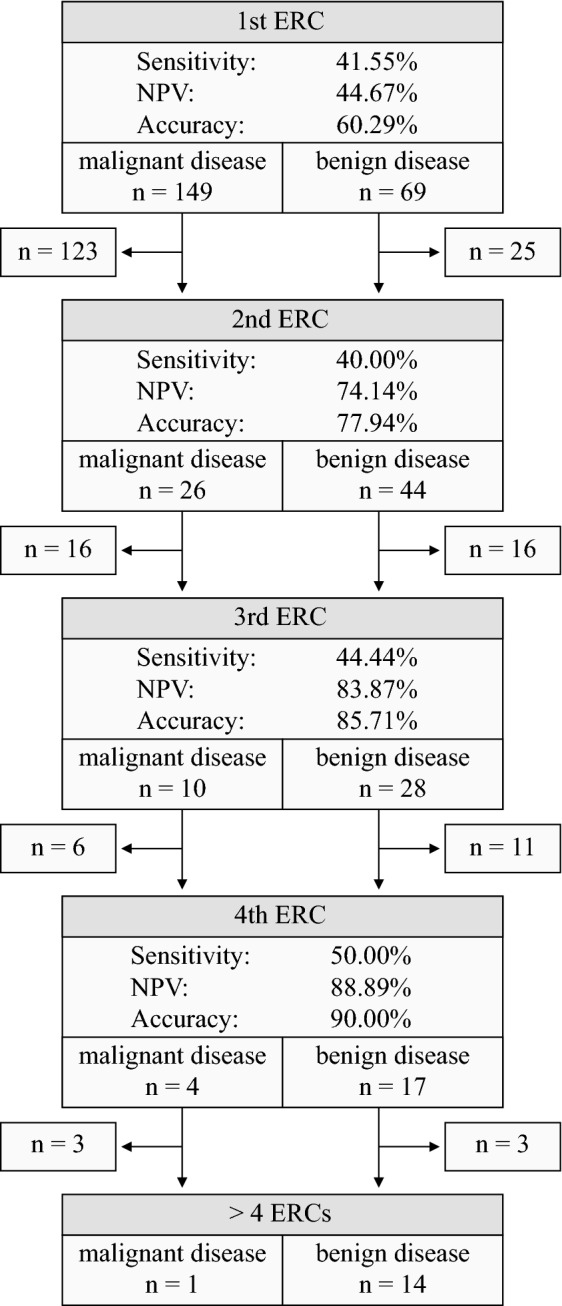
Follow-up ERCs. 70 patients received more than one endoscopic retrograde cholangiography (ERC). Patient drop out (as noted on the sides) was due to the clinical course of malignant disease, intercurrent confirmation of stricture dignity through other modalities or a loss of follow-up. There was only one patient with malignant disease among the 15 patients undergoing more than four ERCs. The denoted sensitivities, negative predictive values (NPV) and accuracies of the ERCs were calculated considering the results of both, brush cytologies and forceps biopsies. Since there were no false-positive diagnoses, specificity and positive predictive values reached 100%.

The underlying diseases are summarized in Table 2. Malign stricture was caused by cholangiocarcinoma (CCA) in 89 cases (59.73%) and by PCA in 38 cases (25.50%). PSC was the most frequent cause of benign stricture with 50.72% (35/69 cases). 40/218 patients (18.35%) fulfilled inclusion criteria for the study due to PSC with dominant stricture (Fig. 2A). In 5 of these cases (12.50%), dominant stricture was caused by a cholangiocarcinoma. 70/218 patients (32.11%), 44 patients with benign and 26 patients with malignant stricture, underwent more than one ERC during the evaluation period (Fig. 3).

**Table 2:** Final diagnosis.

	n	%
**Benign stricture**	69	100
PSC related benign stricture	35	50.72
SSC related benign stricture	4	5.80
IAC related benign stricture	2	2.90
Chronic pancreatitis with benign stricture of the CBD	3	4.35
Recurrent choledocholithiasis with benign stricture	10	14.49
Postoperative benign stricture	1	1.45
Others	8	11.59
Unknown	6	8.70
**Malignant stricture**	149	100
Cholangiocarcinoma	89	59.73
Pancreatic cancer	38	25.50
Gallbladder cancer	5	3.36
Hepatocellular carcinoma	3	2.01
Metastasis of extrahepatobiliary primarius	9	6.04
Others	4	2.68
Unknown	1	0.67

*PSC* primary sclerosing cholangitis, *SSC* secondary sclerosing cholangitis, *IAC* IgG4-associated cholangitis, *CBD* common bile duct.

### Overall diagnostic performance of BC and FB

**Figure 4:**
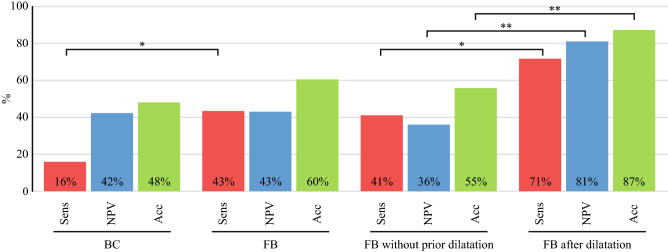
Diagnostic performance of BC and FB. The bar chart opposes the sensitivities (Sens), the negative predictive values (NPV) and the accuracies (Acc) of the respective modalities in percentages (rounded to the nearest whole number). Appendant specificities and positive predictive values were not depicted in this chart, as they were 100% consistently. *FB* forceps biopsy, *BC* brush cytology, *p < 0.05, **p < 0.01.

Altogether 83 brush cytologies and 188 forceps biopsies were obtained during the first ERC with tissue sampling in 218 patients. Sufficient sample material for pathological examination was acquired in 85.54% (71/83 BC) and 97.87% (184/188 FB). Definitely malignant or highly suspicious pathological findings were acquired with BC in 7 of 44 (15.91%) and with FB in 56 of 129 (43.41%) patients with malignant disease (p < 0.01, Fig. 4). Corresponding negative predictive values (NPV) and accuracies were 42.19% and 47.89% for BC and 42.97% (p = 0.92) and 60.33% (p = 0.07) for FB. Specificity and positive predictive values reached 100% for both modalities (no false-positive diagnoses). BC and FB showed higher sensitivities for CCA compared to PCA (CCA: BC: 23.08%, FB: 51.90%, PCA: BC: 10%, FB: 32.35%, CCA vs. PCA: BC: p = 0.65, FB: p = 0.06). The localization of CCA had no significant influence on the sensitivity of FB (distal CCA: 60%, perihilar CCA: 46.15%, intrahepatic CCA: 71.43%, p > 0.05). Due to a low case count in the subgroups, analyses of the sensitivity of BC depending on localization of CCA were not reliable.

### Influence of stricture dilatation on the diagnostic performance of forceps biopsy

Balloon dilatation of the stricture was performed in 49/218 cases (22.48%). FB was performed after prior balloon dilatation in 31 cases and without prior dilatation in 158 cases (Fig. 4). Stricture dilatation to 6–10 mm (depending on the diameter of the bile duct) prior to FB provided a significant diagnostic advantage: Without prior dilatation, FB yielded a sensitivity of 40.52%, an NPV of 35.51% and an accuracy of 55.19% (Fig. 4). After stricture dilatation, sensitivity, NPV and accuracy significantly increased to 71.43% (p = 0.03), 80.95% (p < 0.01) and 87.10% (p < 0.01). In none of the analyzed procedures, balloon dilatation of the stricture was performed prior to BC. Performance of stricture dilatation had a significant influence on the duration of ERC (mean duration of ERC without dilatation: 57.17 ± 24.92 min, mean duration of ERC with dilatation: 69.18 ± 37.37 min, p = 0.04).

### Other potential confounding factors

#### Stricture localization, extent and length

Stricture localization and the respective diagnostic approach are summarized in Table 3. 92.02% (173/188) of the strictures were high-grade with a narrowing > 90%, whereas 7.98% (15/188) of the strictures showed a minor narrowing < 90%. The mean stricture length was 22.45 ± 10.79 mm. Stricture length did not correlate with presence of malignancy (point-biserial correlation coefficient r_pb_ = 0.03). Neither stricture localization nor extent nor length (strictures shorter vs. longer than mean length) significantly influenced the sensitivity of BC or FB.

**Table 3:** Frequencies of the applied modalities for transpapillary tissue sampling depending on stricture localization.

	Stricture localization			
	Distal (n = 87) (%)	Perihilar (n = 84) (%)	Intrahepatic ducts (n = 25) (%)	Others^†^ (n = 22) (%)
BC (n = 83)	27.59 (24/87)	39.29 (33/84)	52 (13/25)	59.09 (13/22)
FB^‡^ (n = 188)	94.25 (82/87)	84.52 (71/84)	72 (18/25)	77.27 (17/22)
**Utilized forceps type for FB depending on stricture localization**^**‡**^				
Large diameter forceps (n = 111)	88.89 (48/54)	79.63 (43/54)	68.75 (11/16)	60 (9/15)
Small diameter forceps (n = 35)	14.81 (8/54)	27.78 (15/54)	37.50 (6/16)	40 (6/15)
SpyBite Biopsy Forceps (Boston Scientific, n = 8)	3.70 (2/54)	5.56 (3/54)	12.50 (2/16)	6.67 (1/15)

*BC* brush cytology, *FB* forceps biopsy.

^†^Including multilocular strictures and such involving the whole common bile duct. ^‡^Forceps type could be determined in 139/188 cases retrospectively. Multiple forceps types were used in 15/139 cases.

#### Forceps types

Large diameter forceps and small diameter forceps were used in 79.86% (111/139) and 25.18% (35/139) of the cases (when FB was conducted and forceps type known), respectively (Table 3). Sufficient sample material for pathological examination was acquired in 99.10% (110/111) and 100% (35/35) of the cases using large and small diameter forceps, respectively. Their diagnostic performance did not differ significantly.

#### Effect of prior EST and prior biliary stenting

119 of 211 (56.40%) patients underwent ERC with endoscopic sphincterotomy (EST) prior to the evaluation period or prior to meeting the inclusion requirements for this study. Prior biliary stenting was done in 71 of 218 (32.57%) patients. Neither prior EST nor prior stenting altered sensitivity of BC or FB significantly.

### Implications of pre-interventional CA 19-9 levels on the diagnostic performance of brush cytology and forceps biopsy

Pre-interventional CA 19-9 levels of patients with malignant disease (4218.72 ± 11,828.27 U/ml) were significantly higher than in patients with benign condition (92.95 ± 365.98 U/ml, p < 0.05; see Table 1). ROC analysis provided a suitable cut-off value of 80 U/ml with a remarkable capacity to predict benign and malignant condition (AUC: 0.85 ± 0.04, sensitivity: 73.91%, specificity: 85.71%, PPV: 93.15%, NPV 55.56%, accuracy 77.17%). When the cut-off value was exceeded, NPV and accuracy of BC and FB decreased significantly (BC: sensitivity: 0% vs. 14.29%, p > 0.05, NPV: 65% vs. 5.26%, p < 0.01, accuracy: 65% vs. 18.18%, p < 0.01; FB: sensitivity: 38.10% vs. 44.07%, p > 0.05, NPV: 64.86% vs. 13.16%, p < 0.01, accuracy: 71.11% vs. 48.44%, p < 0.05; each comparison for CA 19-9 lower vs. higher than 80 U/ml).

#### Adverse events

ERC-induced pancreatitis or cholangitis was observed after 14 of 242 (5.79%) and 3 of 244 (1.23%) ERCs, respectively (Table 4). ERC-induced perforation of the bile duct, severe post-interventional situations (hemorrhage requiring transfusions; requirement of intensive care or surgery) or death did not occur in any case. Stricture dignity, applied modality or stricture dilatation showed no correlation with the incidence of adverse events.

**Table 4:** Incidence of adverse events depending on stricture dignity, applied modality and stricture dilatation.

	Incidence of ERC-induced	
	Pancreatitis (%)	Cholangitis (%)
Total (n = 247^†^)	5.79	1.23
**Depending on stricture dignity**		
Malignant stricture (n = 149)	6.94	1.37
Benign stricture (n = 69)	4.35	0
**Depending on applied modality**		
BC solely (n = 31^†^)	9.68	0
FB solely (n = 113^†^)	4.59	1.80
Large diameter FB only (n = 69^†^)	2.99	2.90
Small diameter FB only (n = 9^†^)	11.11	0
**Depending on stricture dilatation**		
No dilatation (n = 196^†^)	5.76	1.55
With dilatation (n = 51^†^)	5.88	0

*ERC* endoscopic retrograde cholangiography, *BC* brush cytology, *FB* forceps biopsy.

^†^Strictures with unconfirmed dignity included.

## Discussion

In suspected malignant biliary strictures tissue acquisition is usually required, since in most cases imaging alone with CT, MRI or EUS does not lead to a definitive diagnosis, which is albeit essential for initiating an appropriate therapy^8,9^. Furthermore, obtaining a histology in case of biliary malignancy is becoming increasingly important to assess the presence of predictive markers in order to offer a personalized chemotherapy, such as *IDH1* with recently presented positive phase III data in the 2nd line therapy^10,11^.

Endoscopic or percutaneous fine needle biopsy is a viable option in case of a visible mass or bile duct wall thickening^12–14^; but most patients with IBiS suffer from cholestasis or even septic cholangitis and require biliary drainage preferably through ERC. In this situation according to current recommendations^4,6^ transpapillary BC or FB is the primary method to obtain tissue for a cytological or histological diagnosis. While the specificity of both methods is excellent, sensitivity and NPV are limited.

In the present study we analyzed retrospectively a cohort of 218 patients, who underwent ERC with BC and/or FB due to a suspected malignant biliary stricture between January 2010 and April 2018, in order to identify parameters that may impact the sensitivity and NPV of tissue sampling. In a metaanalysis from 2015 (including 9 studies and 730 patients) Navaneethan et al.^15^ reported comparable pooled sensitivities of BC and FB in diagnosing malignant biliary strictures of 45% and 48.1%. Our data confirm this observation in a large and representative real-life cohort with a higher sensitivity of FB (43.4%) compared to BC (15.9%) while the specificity was 100% for both methods. Not surprisingly, we also found higher sensitivities for CCA (BC: 23.08%, FB: 51.90%) than for PCA (BC: 10%, FB: 32.35%). This difference might be explained by the fact that for technical reasons the brush detaches the cell material rather superficially, while the forceps penetrates deeper into the targeted tissue. In many cases with submucosal tumor growth or marked desmoplastic fibrosis and especially in case of an extraluminal tumor like PCA, the cell material obtained by BC might be suboptimal. It should be noted, however, that the significant difference between BC and FB in our cohort was primarily due to the comparatively poor sensitivity of BC, while FB had a sensitivity that was comparable with previously reported ones^15^. This might be partly explained by the fact that we did not routinely use advanced cytologic techniques, such as digital image analysis and fluorescence in situ hybridization, which improve the sensitivity of BC for diagnosing malignancy in IBiS^16–18^. Moreover, we obtained adequate material for cytological examination in only 85.54% with brushing, which might be due to the relative high percentage of very fibrotic PSC related strictures. Other centers, however, reported excellent results of ERC with BC in screening for PSC related biliary malignancy^19^. Furthermore, we have used BC much less frequently, so that a certain selection bias cannot be excluded. It is therefore questionable whether our results should generally lead to the conclusion that FB is superior to BC. Rather, local cytopathological expertise as well as anatomical and technical conditions should also be considered. In order to optimize the cellular yield and thereby increase its sensitivity, some technical improvements of the brush have also been presented in the meantime^20,21^.

Given the limited sensitivity of BC and FB and in order to avoid false negative results, it is recommended either to use an alternative method like EUS-FNB for tissue sampling^5,6^ or to repeat the ERC with FB/BC (supplemented if necessary with advanced imaging techniques like cholangioscopy or intraductal ultrasound). Our findings support the latter recommendation: Taking into account an inevitable patient drop-out due to the clinical course of malignant disease, confirmation of stricture dignity through other modalities or a loss of follow-up, repetition of tissue sampling via ERC led to satisfying NPVs (with the 4^th^ ERC yielding an NPV of 88.89%, see Fig. 3). As we could show, pre-interventional CA 19-9 levels proved to be a valuable tool in order to assess the reliability of negative cytological/histological findings of BC or FB. The NPV of BC and FB decreased significantly when a cut-off value of 80 U/ml was exceeded. Negative cytological/histological findings of BC or FB despite exceedance of the CA 19-9 cut-off value of 80 U/ml should encourage to repeat tissue sampling.

When we retrospectively analyzed a potential impact of other confounding co-variates, we found no significant impact of prior EST or prior biliary stenting on the sensitivity of BC or FB. In contrast to others^22–24^ we could also not confirm a significant impact of stricture length, stricture localization or stricture extent on the sensitivity of transpapillary tissue sampling. Also, sensitivity of FB did not differ between a large or small diameter forceps and the sample yield was excellent with both types. Nevertheless, according to previous publications modification of forceps type^25,26^ might even improve sensitivity or make it easier to insert the forceps into the bile duct and to reach the stricture. The most relevant finding in our study is probably the significantly better sensitivity and NPV of FB following prior balloon dilation (which was primarily indicated in order to secure bile flow and to allow the implantation of a biliary stent). In our study, we performed FB after balloon dilatation in 31 cases and without prior dilatation in 158 cases (Fig. 4). This somewhat more aggressive approach increased the duration of ERC (57.17 ± 24.92 min vs. 69.18 ± 37.37 min, p = 0.04) but was not associated with an increased complication rate such as perforation, bleeding or pancreatitis (Table 4). However, the sensitivity [71.43% vs. 40.52% (p = 0.03)], the NPV [80.95% vs. 35.51% (p < 0.01)] and the accuracy [87.10% vs. 55.19% (p < 0.01)] were significantly higher for FB with prior balloon dilatation compared to FB without prior dilatation (Fig. 4). A potential selection bias cannot be excluded since balloon dilatation of the stricture was performed in only 49/218 cases (22.48%) and more likely in narrow strictures with relevant impairment of the bile flow. However as noted above, stricture extent did not have a significant influence on the diagnostic performance of FB in our study. Cholangiocarcinoma, the most common cause of IBiS in our study, often shows a periductal-infiltrating growth pattern with a dense desmoplastic fibrous stroma^27^. We speculate that balloon dilation tears the superficial desmoplastic and fibrous tissue so that the forceps can reach better and more effectively the submucosal tumor parts. Two previous studies analyzed the impact of stricture dilatation on the diagnostic yield: The first study^28^ on 46 patients reported that a combination of stricture dilation, endoscopic needle aspiration, and biliary brushing significantly improved the diagnostic yield for IBiS; the second study^29^ (139 patients) could not confirm a better sensitivity of BC after dilatation. In the present study we did not perform a balloon dilatation prior BC, because we expected that it is more difficult for the bristles of the brush to scrape cellular material from a stenosis that is then no longer narrowed. Therefore, in contrast to both previous studies, our study is the first which analyzes the effect of prior balloon dilatation on the sensitivity of forceps biopsy.

In conclusion, FB turned out to be the more effective method for transpapillary tissue sampling in our center. Balloon dilatation of the stenosis before FB provided a significant diagnostic benefit and was not associated with more complications. When both, tissue sampling and stricture dilatation, are indicated, we therefore recommend to dilate first and to take the FB afterwards. In order to validate our findings, we would advocate a prospective case control study. For this study design, the recruitment of patients will certainly take several years and could be accelerated by a contribution of multiple centers to the study.

## Data Availability

The datasets used and/or analyzed during the current study are available from the corresponding author on reasonable request.
